# Cuvette-Type LSPR Sensor for Highly Sensitive Detection of Melamine in Infant Formulas

**DOI:** 10.3390/s19183839

**Published:** 2019-09-05

**Authors:** Seo Yeong Oh, Min Ji Lee, Nam Su Heo, Suji Kim, Jeong Su Oh, Yuseon Lee, Eun Jeong Jeon, Hyungsil Moon, Hyung Soo Kim, Tae Jung Park, Guiim Moon, Hyang Sook Chun, Yun Suk Huh

**Affiliations:** 1 Department of Biological Engineering, Inha University, Incheon 402-751, Korea; 2 Electron Microscopy Research Center, Korea Basic Science Institute, Daejeon 34133, Korea; 3 New Hazardous Substances Team, Department of Food Safety Evaluation, National Institute of Food and Drug Safety Evaluation, Ministry of Food and Drug Safety, Cheongju-si 28159, Korea; 4 Department of Chemistry, Chung-Ang University, Seoul 06974, Korea; 5 School of Food Science and Technology, Chung-Ang University, Anseong 17546, Korea

**Keywords:** LSPR, *p*-NA, melamine, infant formula, cuvette-type chip

## Abstract

The globalization of food distribution has made necessary to secure safe products to the general consumers through the rapid detection of harmful additives on the field. For this purpose, we developed a cuvette-type localized surface plasmon resonance (LSPR) sensor that can be easily used by consumers with conventional ultraviolet-visible light spectrophotometer for *in-situ* measurements. Gold nanoparticles were uniformly deposited on a transparent substrate via a self-assembly method to obtain a plasmonically active chip, and the chemical receptor p-nitroaniline (*p*-NA) was functionalized to stabilize the device sensitivity under external temperature and pH conditions. The fabricated chip was fixed onto a support and combined with a cuvette-type LSPR sensor. To evaluate the applicability of this sensor on the field, sensitivity and quantitative analysis experiments were conducted onto melamine as a model sample from harmful food additives. Under optimal reaction condition (2 mM *p*-NA for 20 min), we achieved an excellent detection limit (0.01 ppb) and a dynamic range allowing quantitative analysis over a wide concentration range (0.1–1000 ppb) from commercially available milk powder samples.

## 1. Introduction

In modern society, with the rapid globalization of various cultures and eating habits, the intentional illegal distribution of harmful substances and corrupt foods, which are not allowed for economic gain, is steadily increasing worldwide [1] and this phenomenon is defined as food fraud or economically motivated adulteration (EMA) of food [2]. Because of the importance of food safety, various analytical methods and equipment have been developed to detect harmful substances in food [3]. However, since food is directly transmitted to the end consumer and immediately consumed, there is an urgent need for technologies directly usable by the consumers on the field, i.e., the market, to make quick judgments [4]. Along with the recognition of the importance of on-site detection, several research groups have tried to analyze harmful substances from food and developed, as typical examples, colorimetric assays and lateral flow immunoassays (LFIAs) techniques driven by immune responses [5,6,7]. Nevertheless, to be passed on to general consumers, these on-site detection technologies require the use of simpler and more economical chip fabrication.

In this study, we used the plasmonically active sensor chip developed by our group for the rapid and highly sensitive on-site detection of melamine in infant formulas, which has become a social issue. Melamine (1,3,5-triazine-2,4,6-triamine), which became known during the so-called China milk scandal in 2008, is a trimer of cyanimide and 66% of its mass consists of nitrogen. Due to its high nitrogen content, it has been illegally added to increase the nitrogen amount in food for false high protein concentration despite its non-nutritional nature [8]. Melamine alone has low toxicity but, when combined with a large amount of cyanuric acid, it forms nonaqueous crystals that can result in acute renal toxicity and kidney stone [9]. Therefore, the United States Food and Drug Administration (U.S. FDA) has recommended a tolerable daily intake of 0.63 mg/kg for melamine, set strict guidelines for its analogs, and regulated to not exceed 2.5 ppm in all foods except infant formulas (1 ppm) [10]. Moreover, the Ministry of Food and Drug Safety (MFDS) of South Korea has forbidden the use of melamine in foods for special medical purposes and infant/young children and updated its content limit to 2.5 ppm in other edible products [11]. General techniques used for melamine detection in food include high-performance liquid chromatography (HPLC) [12], gas chromatography/mass spectrometry (GC/MS) [13], and enzyme-linked immunosorbent assay (ELISA) [14,15], but they present some constraints such as long detection times, high costs, and need for expertise [16,17]. To overcome the limitations of conventional analytical techniques, there are ongoing researches on melamine detection systems such as aptamer-based sensors [18], quantum dots-based sensors [19], colorimetric sensors [20], and electrochemical sensors [21] with high sensitivity and on-site applicability.

We have developed a localized surface plasmon resonance (LSPR) sensor using the gold packed glass chip based on the collective oscillation of electrons on the metal nanostructure surrounded by a dielectric [22]. This device can be used as a portable sensor due to its easy accessibility and high potential for multiple sample analysis and miniaturization, which is given by its capability observing the peak change through the existing ultraviolet-visible light (UV-VIS) spectrophotometer without a dedicated detector [23]. In addition, the high sensitivity, low cost, and fast analysis time of the LSPR technology make it still used for detecting biochemical substances in various fields, such as medical diagnosis, food safety control, and environmental pollution monitoring, and allow the development of portable detection devices [24,25].

**Scheme 1:**
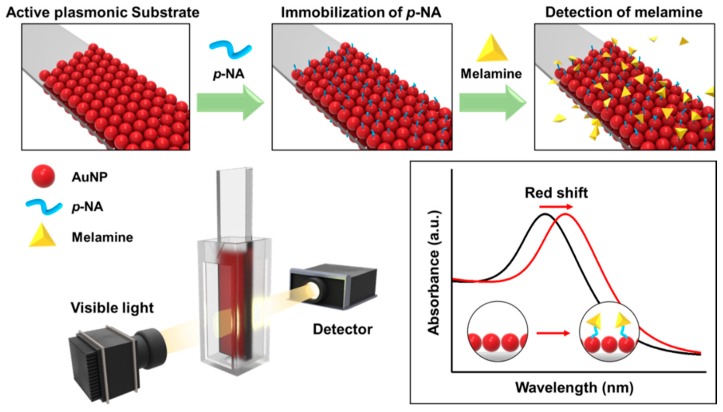
Schematic illustration of the *p*-nitroaniline-based plasmonically active chip for melamine detection.

In this study, we fabricated a plasmonically active chip with high reproducibility and sensitivity by immobilizing uniform gold nanoparticles (AuNPs) at high density on a transparent glass substrate via a self-assembly technique. Then, *p*-nitroaniline (*p*-NA), which is a chemical receptor for melamine [26], was attached to the AuNPs for selective binding with melamine. We optimized the *p*-NA concentration for its bonding stability with the AuNPs and performed UV-VIS spectroscopy measurement to confirm the stability and detection performance of the proposed LSPR sensor (Scheme 1). In addition, to evaluate the possibility in the field, a melamine detection test was conducted on commercial infant formula samples. The LSPR-based technology for on-site melamine detection presented in this study could be used for the fast and reproducible detection of various harmful food additives and contribute to developing a system that guarantees safe food consumption.

## 2. Materials and Methods

### 2.1. Reagents and Instruments

Gold(Ⅲ) chloride trihydrate (≥99.9%), (3-Aminopropyl)-triethoxysilane (≥98.0%) (APTES), *p*-nitroaniline (*p*-NA), bovine serum albumin (BSA), cyanuric acid, urea, uracil, and m-phenylenediamine were purchased from Sigma-Aldrich (St. Louis, MO, USA). Trisodium citrate dihydrate was obtained from Kanto Chemical Co., Inc. (Tokyo, Japan). Methyl alcohol (99.5%) was provided by Samchun Pure Chemical Co., LTD. (Seoul, Korea). Glass substrate from Korea Testing & Research Institute (Gwacheon, Korea) were used. Infant formulas produced by Maeil Dairies Co., Ltd. (Seoul, Korea) were purchased at a local grocery store. Absorbance spectra were recorded on a V-770 UV-VIS spectrophotometer (JASCO International Co., Ltd., Tokyo, Japan), and field emission scanning electron microscopy (FE–SEM) measurements were conducted on a JITACHI S-4300 system.

### 2.2. Synthesis of AuNPs

Prior to fabricating the LSPR sensor chip, 20 nm AuNPs were synthesized. 2.2 mM sodium citrate solution (150 mL) was heated to 100 °C under rapid stirring for 15 min; when the boiling point was reached, 25 mM HAuCl_4_ (1 mL) was added and 3 mL of the resulting solution was collected after 10 min. When the Au seed formed and the temperature dropped down to 90 °C, 25 mM HAuCl_4_ (1 mL) was further added and the mixture was stirred for 30 min. Then, 55 mL of the obtained solution was taken out while distilled water (53 mL) (DW) and a 60 mM sodium citrate solution (2 mL) were added to the remaining solution, which was successively stirred for 20 min. Next, for two consecutive times, further 25 mM HAuCl_4_ (1 mL) was added and the mixture was stirred for 30 min [27]. In order to perform experiments under constant concentration conditions after synthesis of AuNPs, 1.9 × 10^12^ AuNPs/mL was prepared by UV-VIS spectrometer and TEM analysis. 

### 2.3. Fabrication of Plasmonically Active Substrate for the LSPR Chip

The glass substrate (8 mm × 50 mm [width × length]) of the LSPR sensor chip was cleaned by immersion in methanol, 20 min sonication, and rinsing for three times with DW to completely remove the remaining methanol. Then, it was immersed in a 0.5% APTES solution and treated at 50 °C for 1 h; the glass substrate was successively washed five times with DW to remove the remaining APTES solution and dipped in the AuNPs solution (1.9 × 10^12^ AuNPs/mL) for 12 h to allow the nanoparticles to self-assemble as a single layer on it.

### 2.4. Functionalization of p-NA on the Plasmonically Active Substrate

#### 2.4.1. Effect of *p*-NA Concentration on the Plasmonically Active Substrate

The *p*-NA was immobilized on the LSPR substrate for selective melamine detection. First, it was diluted with DW to 0, 0.5, 1, 1.5, 2, and 2.5 mM (500 μL); each solution was reacted with a plasmonically active substrate for 20 min by dipping and then washed away with DW. The *p*-NA immobilized LSPR substrate was then blocked with 3% (w/v) BSA (500 μL) for 10 min by dipping. The *p*-NA-functionalized chip was analyzed for the absorption spectrum of AuNPs by using a UV-Vis spectrophotometer. The resolution of the UV-VIS spectrophotometer was 0.025–5 nm, and the resolution of the spectral results obtained in this study was 0.05 nm.

#### 2.4.2. Effect of *p*-NA Reaction Time on the Plasmonically Active Substrate

To determine the optimal time for the sufficient *p*-NA immobilization on the LSPR chip, the *p*-NA solution (2.0 mM) was reacted with the LSPR chip over different times (0, 5, 10, 20, 30, and 40 min) and unbound *p*-NA was washed away with DW. The change in the plasmonic band of the fabricated chips was analyzed by UV-VIS spectroscopy.

### 2.5. LSPR Sensing of Melamine in Distilled Water

The melamine detection performance of the LSPR sensor fabricated under optimal *p*-NA (2.0 mM, 20 min) and 3% BSA condition was evaluated using melamine solutions in DW. *p*-NA-based LSPR sensor was dipped for 20 min in a melamine solution at various concentrations. Then, the sensor was washed with DW to remove melamine not bonded with *p*-NA and the absorption spectrum was recorded on the UV-VIS spectrophotometer. To verify the detection limit of this sensor, additional samples were prepared by diluting samples with 0, 0.01, 0.1, 1, 10, 100, and 1000 ppb, respectively.

### 2.6. Melamine Detection in Infant Formulas

Based on the detection results for the melamine-spiked solutions in DW, several experiments were conducted to confirm the sensor capability of melamine detection in actual milk powder. First, melamine was added to milk powder (100 mg/mL) purchased from a local grocery store and diluted to the final concentrations of 0, 0.01, 0.1, 1, 10, 100, and 1000 ppb. Then, the melamine added milk powder was centrifuged at 10,000 rpm for 10 min and the supernatant was collected. Each sample was loaded with a *p*-NA-based LSPR sensor for 20 min and analyzed with the UV-VIS spectrophotometer.

### 2.7. Selective Detection of Melamine

To confirm the selective binding between *p*-NA and melamine, a selective detection experiment was conducted by using the proposed *p*-NA-functionalized LSPR sensors. Melamine was diluted to 0.01 ppb in DW. Cyanuric acid, uracil, urea, and m-phenylenediamine were separately diluted to 100 ppb in DW. Each sample was reacted with a LSPR sensor chip functionalized with 2.0 mM *p*-NA for 20 min and, then, analyzed by using the UV-VIS spectrophotometer.

## 3. Results

### 3.1. Fabrication of Plasmonically Active Substrate for the LSPR Chip

**Figure 1:**
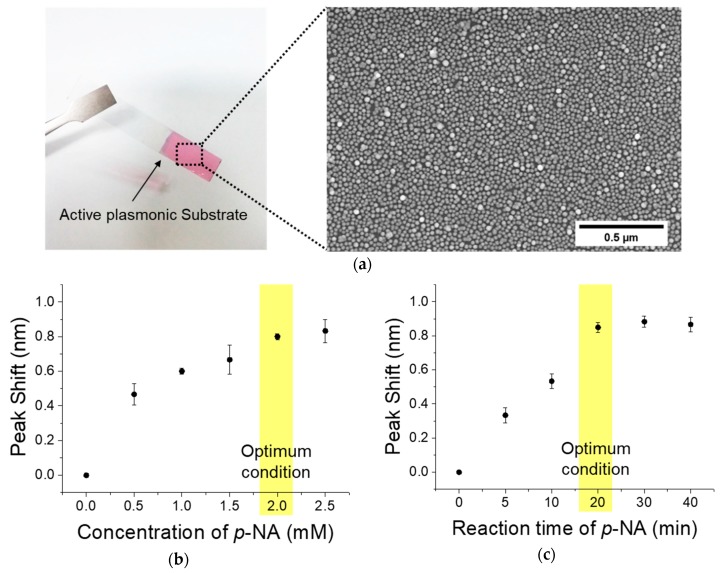
Optimum conditions for melamine detection by *p*-NA-based localized surface plasmon resonance sensor chip. (**a**) Optical (left) and scanning electron microscopy (right) images of an optimized plasmonically active substrate. Effect of the *p*-NA (**b**) concentrations and (**c**) immobilization time on the plasmonically active substrate. All experiments were performed with five measurements, and the data represent mean ± standard deviation.

Since the proposed plasmonically active chip is influenced by the refractive index of the dielectric environment surrounding the nanoparticles, the technology to uniformly and stably deposit metal (Au or Ag) nanoparticles on a transparent glass substrate is an important factor in the development of reproducible LSPR sensors. The metal nanoparticles used for plasmonic sensing are usually made of gold or silver. Since Ag particles result in a narrower and stronger LSPR peak and lower chemical stability compared to the case of gold, AuNPs are mainly adopted in biosensors and they were also used in this study. Highly sensitive LSPR sensors were fabricated by deposition of densely packed AuNPs on a transparent substrate. In brief, the surface of glass substrate to be used as the plasmonically active chip was washed with ultrasound and methanol and, successively, functionalized with amino groups for 1 h at 50 °C by using APTES. Then, the unbound APTES was removed by washing with DW five times and the plasmonically active substrate was prepared by dipping in the AuNPs solution for 12 h. In these processes, the negatively charged AuNPs would be deposited by electrostatic interaction with amino groups on the functionalized glass substrate. As shown in the optical image in Figure 1a, the plasmonic chip was designed to be fixed to the UV cuvette cell to allow its easy testing with a commercially available UV-VIS spectrophotometer. To ensure the reproducibility and sensitivity of the proposed cuvette-type LSPR chip, we confirmed the deposition state of the AuNPs by scanning electron microscopy (SEM) and UV-VIS spectroscopy; the SEM analysis revealed that the AuNPs were uniformly and densely deposited on the glass substrate. Reliability of the melamine detection test confirmed that all the fabricated chips satisfied the conditions of specific plasmon wavelength and intensity of AuNPs in the DW solution and, then, proceeded to the study of the subsequent process.

### 3.2. p-NA-Functionalization of the Plamonically Active Chip

For selective detection of melamine, chemoreceptor *p*-NA was selected. We have developed an economical sensor technology that can be applied in the field using a long-term stable chemoreceptor under external environmental conditions. The *p*-NA chemical receptor is not only known to bind specifically to melamine but also has an advantage property for functionalizing on AuNPs. In the *p*-NA structure, benzene is *para*-substituted with an amine and a nitro group; the amine group can be easily combined with AuNPs having a negative surface charge, without any linker. Melamine, which is rich in electrons, an electron donor and both melamine and *p*-NA can be selectively bound while the nitro group of *p*-NA acts as an electron acceptor.

To functionalize the *p*-NA on the plasmonically active substrate, we experimentally optimized the change in the plasmon wavelength of the AuNPs according to the chemical receptor concentration and the reaction time (Figure 1b,c). The *p*-NA capable of detecting melamine was coupled to a LSPR chip immobilized with AuNPs. First, it was functionalized on the AuNPs of the LSPR chip while changing its concentration from 0.0 to 2.5 mM; the surface plasmon band of the AuNPs was continuously red-shifted by increasing *p*-NA concentration and this shifting width decreased above 2.0 mM (Figure 1b). The final *p*-NA concentration of 2.0 mM, with a small error rate, was determined at the *p*-NA concentration condition, which showed excellent peak shifting. Next, the binding force between chip and *p*-NA was investigated with the reaction time up to 40 min (Figure 1c); the peak shift value gradually increased with the reaction time until 20 min and, then, did not change until 40 min. Based on these experimental results, the optimal *p*-NA concentration and the reaction time were determined as 2.0 mM and 20 min, respectively.

### 3.3. Melamine Detection by the Cuvette-Type LSPR Sensor

**Figure 2:**
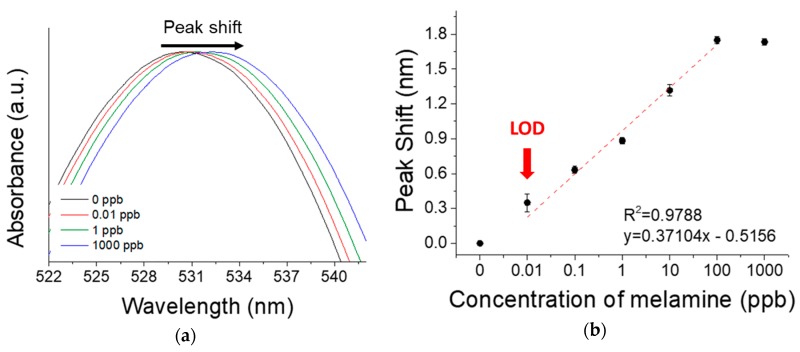
Melamine detection by *p*-NA-based cuvette-type LSPR sensor. Peak (wavelength) shift for different melamine concentrations (0–1000 ppb). (**a**) The change of the plasmon spectrum and (**b**) the linear correlation with the melamine concentration in standard solution. All experiments were performed with five measurements, and the data represent mean ± standard deviation. The coefficient of variation (% CV) is below 10%.

In this study, we have developed a LSPR sensor that can quantify the melamine concentration in milk powder without expensive and time-consuming conventional equipment. The detection limit of melamine was measured using a cuvette-type LSPR sensor prepared from 0.01 to 1000 ppb. Figure 2a showed the change of the plasmon spectrum with the melamine concentration; as expected, it was red-shifted when increasing the concentration. In order to quantitatively analyze these results, we plotted again the peak shift values as a function of the concentration change (Figure 2b), finding that the peak shift value was linearly correlated with the melamine concentration increase up to 100 ppb. The minimum detectable value was derived from the dynamic range, represented by the linear correspondence region in Figure 2b, and revealed that the developed LSPR sensor achieved a detection limit of 0.01 ppb. To prove the reliability of our LSPR sensor for melamine detection, we confirmed the reliability by calculating the coefficient of variation (%CV). The %CV obtained from this LSPR sensor was lower than 10% (satisfactory precision) [28], and the %CV should not exceed 20% after 10 replicates of a sample [29]. Based on these results, melamine detection using this LSPR sensor is considered to be a reproducible sensing method.

### 3.4. Selective Detection in Melamine-Spiked Infant Formulas

**Figure 3:**
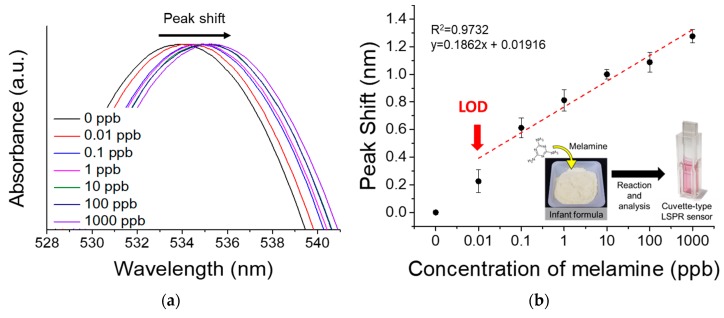
Specific melamine detection in infant formulas. (**a**) The change of the plasmon spectrum and (**b**) the linear correlation with the melamine concentration in melamine-spiked infant formulas. All experiments were performed with five measurements, and the data represent mean ± standard deviation. The coefficient of variation (% CV) is below 10%.

Based on the results for the melamine standard solutions, melamine detection tests were performed on commercial milk powder (Figure 3) to evaluate the applicability of the LSPR sensor. The samples were prepared by adding different concentrations (0.01–1000 ppb) of melamine to the milk powder (Figure 3a). The detection results were similar to those for the standard solutions; however, the peak shift of the plasmon spectrum when increasing the melamine concentration was smaller, while the linear response interval showed a wider dynamic range of 0.01–1000 ppb (Figure 3a,b). Different peak shifts were noticed when testing infant formula samples, which could be because of the matrix effect [30]. Comparative analysis of melamine detection techniques in Table 1 showed that the *p*-NA-based LSPR sensor developed in this study had better detection sensitivity than other reported methods.

**Figure 4:**
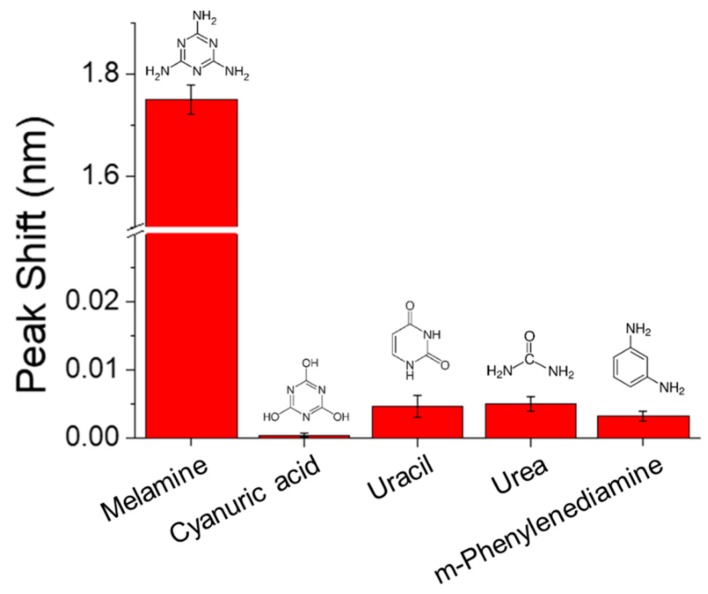
Melamine selectivity of *p*-NA-based cuvette-type LSPR sensor from DW additionally containing various harmful additives such as melamine, cyanuric acid, uracil, urea, and m-phenylenediamine. The concentration of melamine and the other substances are 100 ppb. All experiments were performed with five measurements, and the data represent mean ± standard deviation. The coefficient of variation (% CV) is below 10%.

In addition, the selective melamine detection was assessed by adding other harmful additives such as cyanuric acid, uracil, urea, and m-phenylenediamine to the infant formula. As shown in Figure 4, a peak shift of about 2–4 times was observed in the melamine sample due to the excellent selective binding property of *p*-NA. From these results, we concluded that the *p*-NA-based LSPR sensor can be used to selectively detect melamine in actual milk powder down to, at least, 0.01 ppb.

## 4. Conclusions

In this study, we fabricated a transparent plasmonically active substrate with AuNPs uniformly and densely deposited on it and developed a highly sensitive LSPR sensor that can quickly detect melamine, a harmful food additive. The plasmonically active substrate was prepared by modifying amino groups with 0.5% APTES for 12 h and, then, uniformly depositing AuNPs via a self-assembly method. To develop a LSPR sensor capable of detecting melamine directly on site, i.e., the market, we functionalized *p*-NA, which is a selective chemical receptor for melamine and stable under external temperature and pH conditions. Based on the optimized conditions for functionalizing *p*-NA (2.0 mM *p*-NA for 20 min) on the plasmonically active substrate, we fabricated a cuvette-type LSPR sensor for melamine detection. The minimum detection limit achievable by this LSPR sensor chip was evaluated using both standard melamine solutions and melamine-spiked milk powder ones. The dynamic range (0.01–1000 ppb) for the quantitative analysis of melamine was wider for the actual milk powder samples than for the standard ones and the corresponding limit of detection was 0.01 ppb. Therefore, our *p*-NA-based LSPR sensor chip could be used for the screening of contaminated foods and agricultural products, in addition to milk powder. Its manufacturing in the cubic shape allows high sensitivity and low cost. Such cuvette-type LSPR sensor chip can be easily utilized through coupling with conventional UV-VIS spectrometer and facilitates economic chip manufacturing using existing cuvette cell process. This is a promising, fast, and accurate next-generation analytical technology; the cost-effective self-assembly process adopted in this work could be utilized for next-generation detection technologies because of the low mass production cost and excellent sensitivity.

## Figures and Tables

**Table 1:** Limits of detection (LODs) of various melamine analytical methods in infant formula.

Method	Comment	Receptor	LOD	Linear Range	Reference
Colorimetry (AuNPs)	Simple, sensitive, low cost, on-site applicability	1-(2-mercaptoethyl)-1,3,5-triazinane-2,4,6-trione (MTT)	2.5 ppb		[31]
Colorimetry (AgNPs)		*p*-nitroaniline (*p*NA)	0.1 ppm		[26]
HPLC	Complex, expensive, time-consuming, and sensitive		0.1 ppm	1.0–80 ppm	[12]
SPR		MEL-Ab	1.4 ppb	1.4–172 ppb	[32]
SERS		4-mercaptopyridine (MPY)	0.1 ppb	0.5–100 ppb	[33]
FTIR/NIR	Low sensitivity		1 ppm		[34]
LSPR	Simple, high sensitivity, low cost, and on-site applicability	*p*-NA	0.01 ppb	0.01 ppb–1000 ppb	This work

## Supplementary Files

Supplementary File 1
