# Erratum: Cuvette-Type LSPR Sensor for Highly Sensitive Detection of Melamine in Infant Formulas. *Sensors* 2018, *19(18),* 3839

**DOI:** 10.3390/s19214630

**Published:** 2019-10-24

**Authors:** Seo Yeong Oh, Min Ji Lee, Nam Su Heo, Suji Kim, Jeong Su Oh, Yuseon Lee, Eun Jeong Jeon, Tae Jung Park, Hyang Sook Chun, Yun Suk Huh

**Affiliations:** 1 Department of Biological Engineering, Inha University, Incheon 402-751, Korea; seoyeong1001@gmail.com (S.Y.O.); dlalswlcjsfl@gmail.com (M.J.L.); bioheo6@gmail.com (N.S.H.); rlatnwl2007@gmail.com (S.K.); ojssjo0620@naver.com (J.S.O.); dbtjs2226@naver.com (Y.L.); 0914dms@naver.com (E.J.J.); 2 Electron Microscopy Research Center, Korea Basic Science Institute, Daejeon 34133, Korea; 3 Department of Chemistry, Chung-Ang University, Seoul 06974, Korea; tjpark@cau.ac.kr; 4 School of Food Science and Technology, Chung-Ang University, Anseong 17546, Korea

The authors wish to make the following erratum to this paper [1]:

**Request to exclude author names affiliated with Ministry of Food and Drug Safety.** The following authors belonging to a Korean government institute had to participate in this study with the approval of the Ministry of Food and Drug Safety (MFDS) before the paper was published. In this regard, the following authors have been warned by the MFDS to exclude their names as authors of this paper. All authors discussed this issue together, and all agreed to exclude the names of the authors below. We apologize for any inconvenience this may have caused in omitting the official research procedure of the MFDS. We would appreciate if you accept our request.

**Affiliation:** New Hazardous Substances Team, Department of Food Safety Evaluation, National Institute of Food and Drug Safety Evaluation, Ministry of Food and Drug Safety, Cheongju-si 28159, Republic of Korea.

**Authors:** Hyungsil Moon, Hyung Soo Kim, Guiim Moon.

The authors would like to apologize for any inconvenience caused to the readers by these changes.
