# Supplementary material for: Cuvette-Type LSPR Sensor for Highly Sensitive Detection of Melamine in Infant Formulas
Source: Sensors (Basel). 2019 Sep 5;19(18):3839. doi: 10.3390/s19183839 (PMC6766901; doi:10.3390/s19183839)
Supplement: Supplementary File 1 [file sensors-19-03839-s001.pdf]

# Supporting Information

# Cuvette-type LSPR Sensor for Highly Sensitive Detection of Melamine in Infant Formulas

Seo Yeong Oh[1,†], Min Ji Lee[1,†], Nam Su Heo[1,2,†], Suji Kim[1], Jeong Su Oh[1], Yuseon Lee[1], Eun Jeong Jeon[1], Hyungsil Moon[3], Hyung Soo Kim[3], Tae Jung Park[4], Guiim Moon[3,*], Hyang Sook Chun[5,*], Yun Suk Huh[1,*]

[1]Department of Biological Engineering, Inha University, Incheon 402-751, Republic of Korea

[2]Electron Microscopy Research Center, Korea Basic Science Institute, Daejeon 34133, Republic of Korea

[3]New Hazardous Substances Team, Department of Food Safety Evaluation, National Institute of Food and Drug Safety Evaluation, Ministry of Food and Drug Safety, Cheongju-si, 28159, Republic of Korea

[4]Department of Chemistry, Chung-Ang University, Seoul 06974, Republic of Korea

[5]School of Food Science and Technology, Chung-Ang University, Anseong 17546, Republic of Korea

† *These authors have contributed equally to this work.*

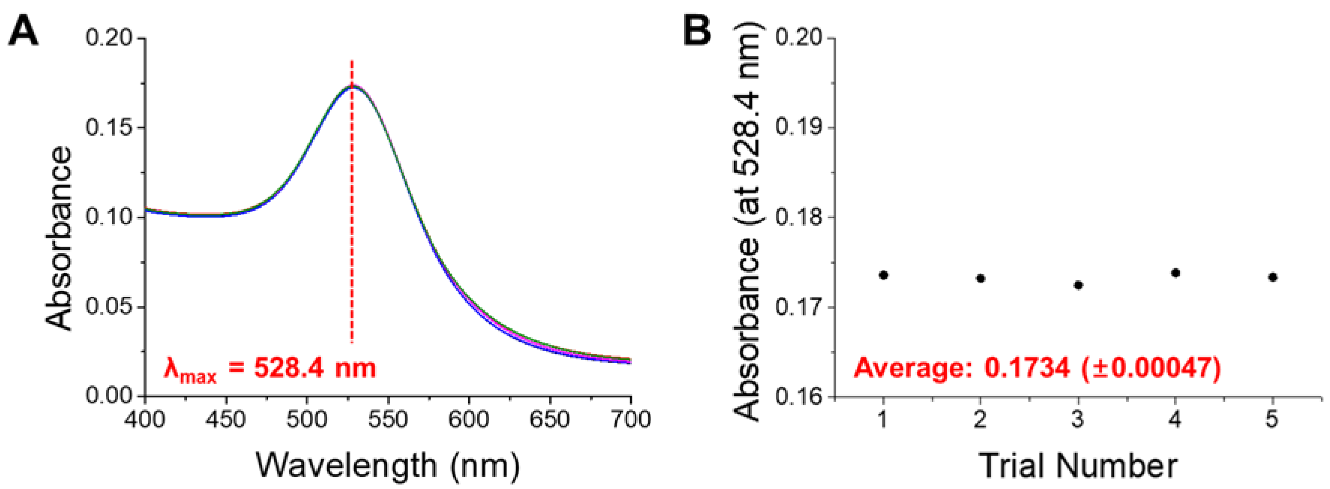


**Figure S1.** Reproducibility of LSPR sensor chip fabricated by assembling plasmonic chip deposited with AuNPs. (A) Absorbance spectrum of five LSPR sensor chips and (B) measured absorbance averages (0.1734 ± 0.00047) at 528.4 nm.

As shown in Figure S1, it was confirmed that the absorbance spectrum and absorbance values of LSPR chips fabricated in this study were consistently reproducible. A plasmonic substrate having an absorbance value ($A_{528.4\ nm}$) of 0.1734 (± 0.00047) at 528.4 nm, the maximum absorption wavelength of the synthesized AuNPs, was used. Thereafter, the experiments were performed by assembling the LSPR chip using a plasmonic substrate having a constant absorbance value.

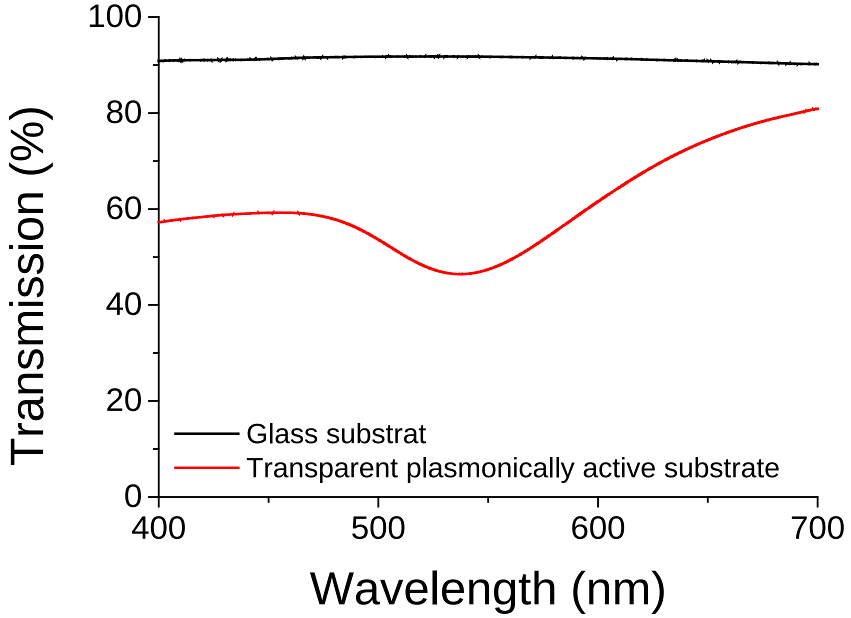


**Figure S2.** UV/Vis Spectra of glass substrate before and after the deposition of AuNPs on a transparent substrate.

Figure S2 showed the UV/Vis spectrum before and after AuNPs deposition of the transparent substrate used in this study. As shown in Figure S2, the bare glass substrate showed more than 90% transmittance in the wavelength range of 400–700 nm, and the plsmonically active substrate showed more than 55% transmittance except for the plasmon absorption region of AuNPs peak.

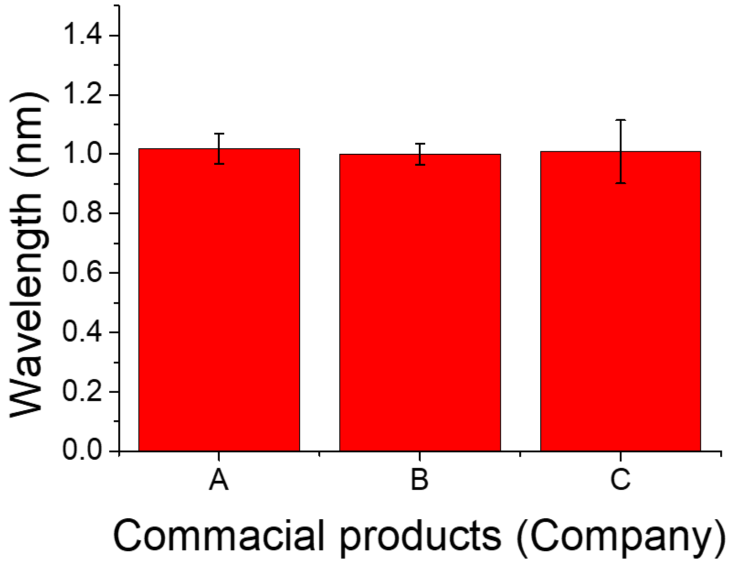


**Figure S3.** Selective LSPR melamine detection from three infant formulas on the market. Melamine was added to three commercial infant formulas to prepare each final 10 ppb melamine-spiked infant formula sample (The three Korean milk powder manufacturers used in this study were (A) Namyang Dairy, (B) Maeil Dairy and (C) Ildong Dairy).

In addition, in order to reconfirm the results of melamine detection in infant formula, two additional milk powders, including the milk powder used in this study, were purchased, and further selective detection experiments were conducted. Using a total of three milk powders on the market, melamine-spiked infant formula samples were prepared for selective melamine detection. As shown in Figure S3, melamine was selectively detected with similar detection sensitivity regardless of three milk powder manufacturer samples in South Korea. Through these results, we confirmed that the *p*-NA functional LSPR sensor developed in this study can selectively detect melamine from commercial infant formula.

**Table S1.** Comparison with *p*-NA and aptamer for melamine detection.

| Method | Receptor | | LOD | Linear Range | Reference |
|---|---|---|---|---|---|
| AuNPs, Colorimertry, in Milk | Aptamer | 32 T | 1.88 ppb | 0-63.1 ppb | [1] |
| | | 31 T | 4.29 ppb | 12.6-126.1 ppb | [2] |
| | | 10 T | 5.26 ppb | - | [3] |
| AuNPs, LSPR, in Infant Formula | *p*-NA | | 0.01 ppb | 0.01-1,000 ppb | This work |

For the comparative analysis of *p*-NA, a chemoreceptor used in this study, and aptamer, a bio-receptor, we prepared a comparative table of melamine sensor references based on aptamers (Table S1). The aptamers used for the detection of melamine in milk are mainly composed of thymine bases, which are known to show selective hydrogen bonding properties with melamine [1-3]. Research reports on the detection of melamine using aptamers from infant formula have not been confirmed to date. Aptamers generally have the advantage of superior selectivity, but they are more expensive than chemoreceptors and have limitations in that they are not stable to external environmental conditions. In addition, the LSPR application of this study requires additional functionalization (thiol-modification) to combine AuNPs and Aptamers. On the other hand, *p*-NA used in this study can be combined with AuNPs only by dipping process, and has excellent sensitivity and wide dynamic range. For these reasons, we conducted the experiments by selecting *p*-NA as a chemoreceptor for the detection of melamine from infant formula.

**References**

1. Hu, X.; Chang, K.; Wang, S.; Sun, X.; Hu, J.; Jiang, M., Aptamer-functionalized AuNPs for the high-sensitivity colorimetric detection of melamine in milk samples. *PLOS ONE* **2018**, 13, (8), e0201626.

2. Xing, H.; Zhan, S.; Wu, Y.; He, L.; Zhou, P., Sensitive colorimetric detection of melamine in milk with an aptamer-modified nanogold probe. *RSC Advances* **2013**, 3, (38), 17424-17430.

3. Huang, H.; Li, L.; Zhou, G.; Liu, Z.; Ma, Q.; Feng, Y.; Zeng, G.; Tinnefeld, P.; He, Z., Visual detection of melamine in milk samples based on label-free and labeled gold nanoparticles. *Talanta* **2011**, 85, (2), 1013-1019.
